# Physiotherapy management in eating disorders

**DOI:** 10.1186/2050-2974-2-S1-P8

**Published:** 2014-11-24

**Authors:** Kristy Chase

**Affiliations:** 1 Flinders Medical Centre, Adelaide, Australia

## 

The Paediatric Unit at Flinders Medical Centre in South Australia launched an Eating Disorder Program in February 2013. The presentation of an eating disorder patient varies among consumers, and treatment requires a sustained, flexible and multidisciplinary approach. Physiotherapy was asked to be involved in the multi-disciplinary team to deliver an inpatient exercise class to provide education on posture and provide a safe environment for healthy exercise. The class began and consisted of two half an hour sessions a week. To increase the knowledge of Physiotherapy management in Eating Disorders a study tour was undertaken to observe the role of physiotherapists at the leading children's hospitals. The hospitals visited include Westmead Children's Hospital, Princess Margaret Hospital and the Starship Children's Hospital in Auckland. On completion of the study tour it was identified that to progress Physiotherapy involvement with the Eating Disorder population we should incorporate an initial physiotherapy assessment when new patients are admitted, provide discharge information for exercise and an information sheet for parents for compulsive exercise. It was also identified that there were no guidelines for Physiotherapy Management in Eating Disorders and this would be beneficial to standardise the role of physiotherapy across Australia.

